# Development of Amphotericin B Micellar Formulations Based on Copolymers of Poly(ethylene glycol) and Poly(ε-caprolactone) Conjugated with Retinol

**DOI:** 10.3390/pharmaceutics12030196

**Published:** 2020-02-25

**Authors:** Yeimy J. Rodriguez, Luis F. Quejada, Jean C. Villamil, Yolima Baena, Claudia M. Parra-Giraldo, Leon D. Perez

**Affiliations:** 1 Grupo de Investigación en Macromoléculas, Departamento de Química, Facultad de Ciencias, Universidad Nacional de Colombia-Sede Bogotá, Carrera 45 No. 26-85, Edificio 451 of. 449, Bogotá D.C. 11001, Colombia; yjrodriguezmo@unal.edu.co; 2 Unidad de Proteómica y Micosis Humanas, Grupo de Enfermedades Infecciosas Departamento de Microbiología, Facultad de Ciencias, Pontificia Universidad Javeriana, Carrera 7 No. 43-82, Bogotá D.C. 110231, Colombia; lquejada@javeriana.edu.co (L.F.Q.); jcvillamilp@unal.edu.co (J.C.V.); 3 Grupo de Investigación SILICOMOBA, Departamento de Farmacia, Facultad de Ciencias, Universidad Nacional de Colombia-Sede Bogotá, Carrera 45 No. 26-85, Edificio 451 of. 449, Bogotá D.C. 11001, Colombia

**Keywords:** Amphotericin B, polymer micelles, drug delivery

## Abstract

Amphotericin B (AmB) is a broad spectrum of antifungal drug used to treat antifungal diseases. However, due to the high toxicity of AmB, treated patients may suffer the risk of side effects, such as renal failure. Nanoencapsulation strategies have been reported to elicit low toxicity, albeit most of them possess low encapsulation efficiency. The aim of this research is to develop micellar delivery systems for AmB with reduced toxicity while maintaining its affectivity by employing retinol (RET)-conjugated amphiphilic block copolymers (ABCs) as precursors. Copolymers composed of poly(ε-caprolactone) (A) and polyethylenglycol (B) of types AB and ABA were synthesized by ring opening polymerization and subsequently conjugated with RET by Steglich esterification. ^1^H-NMR spectroscopy was used to corroborate the structure of copolymers and their conjugates and determine their molecular weights. Analysis by gel permeation chromatography also found that the materials have narrow distributions. The resulting copolymers were used as precursors for delivery systems of AmB, thus reducing its aggregation and consequently causing a low haemolytic effect. Upon conjugation with RET, the encapsulation capacity was enhanced from approximately 2 wt % for AB and ABA copolymers to 10 wt %. AmB encapsulated in polymer micelles presented improved antifungal efficiency against *Candida albicans* and *Candida auris* strains compared with Fungizone^®^, as deduced from the low minimum inhibitory concentration.

## 1. Introduction

During the last decades, the number of people susceptible to the invasive fungal disease (IFD) has increased. It is a consequence of advances in medicine that have increased the life expectancy of HIV-infected patients and augmented the number of procedures, such as organ and bone marrow transplants. Furthermore, the population growth also augments the occurrence of premature births and diseases (i.e., cancer) that can compromise the immune system [1,2]. Accelerated environmental deterioration, extreme weather conditions and limited access to the health system also contribute to the incidence [3]. The diagnosis and treatment of IFD is difficult in many cases due to the minimal specificity of its symptomatology, especially since the identification of the pathogen source has further become complex, mainly in hospital centres where technological resources are unavailable or do not employ a personnel trained for this purpose; resorting to empirical therapies, which implies the use of broad spectrum antifungal drugs [4].

Since 1959, Amphotericin B (AmB) has been the “gold standard” for the treatment of IFDs. Fungizone^®^ (a colloidal dispersion of this drug is stabilized by sodium deoxycholate as a surfactant) is the most widely used formulation of AmB due to its effectiveness, wide spectrum of action and low number of reports of resistant strains [5,6,7]. However, the toxicity of AmB and its bioaccumulation in organs, such as liver, lungs and kidneys, are the undesirable features that seriously affect the treated patients [8,9]. According to a study by Wingard et al. [10], the probability of patients receiving the above medication to suffer from kidney damage could be so high as 70%, indicating both an increase in mortality rate and cost of treatment [11]. With the development of liposomal formulations, among them Ambisome^®^, the toxicity of AmB has been attenuated due to the small size of the liposomes that allows for a prolonged circulation and distribution into the organs. Additionally, the strong interaction between AmB and the liposome components endow a controlled release to the drug [12]. However, high cost apart from high dose (5–10 mg/kg for Ambisome^®^ compared with 0.5–1 mg/kg for Fungizone^®^) requirements are disadvantageous, principally among low- and middle-income countries, such as Colombia [13,14,15,16,17].

Although AmB has been used for 60 years, a debate persists about the mechanism for its antifungal action. The firstly and most accepted model is the formation of barrel-type pores that permeate the double-layer fungus cell membrane. According to this model, the heptaene segments are oriented towards the outside of the pore, while the hydroxyl groups are arranged towards the inside, allowing the cell to lose ions and polar molecules which are vital for its functioning. Pore formation is mediated by the complexation of membrane sterols with AmB molecules [18]. Other mechanisms, such as sequestering of sterols by aggregates of AmB on the surface of cells (sterol sponge) [19], and the formation of reactive nitrogen and oxygen species promoted by AmB that cause irreversible damages to cell integrity, has also being proposed [9].

AmB, despite being an active antifungal drug, is a highly cytotoxic substance, particularly against human red blood cells (RBCs) [20]. This fact is also attributed to its ability to induce cholesterol complexation, which is a component of cell membranes. AmB has a structure resembling ergosterol and therefore has a similar binding pathway. According to simulations by molecular dynamics and density functional theory, the interaction of AmB with sterol occurs through van der Waals forces, π–π interactions and hydrogen bonds [21]. However, an experimental probe found that the interaction of AmB with ergosterol is stronger than that with cholesterol [22]. The preference of AmB for ergosterol, with their structural similarities, is attributed to the high degree of unsaturation, especially to conjugate ring B, which increases the magnitude of π–π interactions with heptane segments of AmB and the van der Waals forces that depend on molecular geometries. A research also suggests that complex formation is less entropically favoured given the high structural flexibility of cholesterol [23].

AmB, with its amphiphilic nature, aggregates when its concentration exceeds its critical aggregation concentration (CAC) of approximately 1 µg/mL [24]. Past research proved that AmB, which occurs in monomeric form in low concentrations, has a high tendency to interact with ergosterol-rich membranes, but cholesterol-containing membranes are unaffected [25]. However, AmB also aggregates and acts as sponge to sequester cholesterol [24]. The presence of AmB water-soluble aggregates induce haemolytic effects on human and mouse erythrocytes; meanwhile, the formulations dominated by the monomeric form have low cytotoxicity [26].

Nowadays, polymer micelles (PMs) have emerged as one the main carriers in the development of nanostructured drug formulations [27,28]. These nanoparticles are composed of a hydrophobic core which is capable of encapsulating water-insoluble drugs such as AmB, and a hydrophilic shell that confers colloidal stability and compatibility with biological systems [29,30]. A recent report indicates that upon administration, PMs dissociate, due to their dilution and interaction with plasmatic constituents such as proteins, producing individual block copolymer chains which are quickly sequestered by Kupffer cells into the liver [31]. It suggests that, in addition to the controlled release of the cargos, PMs are safe nanocontainers for nephrotoxic drugs such as AmB. This is in concordance with in vivo studies which corroborate that micellar systems composed of polystyrene-block-polyethylene oxide [32], linoleic acid modified polyethylene glycol-block-polyethylenimine [33], polyethylene glycol-block-poly(*N*-hexyl stearate l-aspartamide) [34] and partially benzylated poly-L-aspartic acid [35] endow AmB lower toxicity.

The aim of this study is to obtain micellar vehicles for AmB with improved encapsulation efficiency, control the release of the drug and lessen its cytotoxic effect without altering its antifungal effectivity. Amphiphilic diblock and triblock copolymers composed of segments of poly(ethylene glycol) (PEG) and poly(ε-caprolactone) (PCL), which are biodegradable and biocompatible polymers and widely used in biomedical applications, were initially synthesized [36,37,38]. These materials were subsequently conjugated with retinol (RET), which is a hydrophobic biomolecule with a polyenic segment, and evaluated as vehicles for AmB. The resulting formulations were characterized to determine the aggregation state of the drug, its release kinetics, cytotoxicity and antifungal activity.

## 2. Materials and Methods

### 2.1. Materials

ε-Caprolactone (CL) 98%, methoxy-polyethylene glycol (mPEG) of 5 kDa and polyethylene glycol diol (PEG-diol) of 6 kDa, RET (95%), pyrene (99%), AmB (98%), 4-(dimethylamino)pyridine (DMAP) (99%), Tin(II) 2-ethylhexanoate (Sn(Oct)_2_) (95%), succinic anhydride (≥99%), *N*,*N*′-dicyclohexylcarbodiimide (DCC), triethylamine (TEA), resazurin sodium salt, ethylenediaminetetraacetic acid (EDTA), Methylthiazolyldiphenyl-tetrazolium bromide (MTT), Dulbecco’s Modified Eagle’s Medium (DMEN) and other reagents and solvents used in the protocols of synthesis, purification and characterization were purchased from Sigma-Aldrich, St. Luis, MO, USA. Fungizone^®^ manufactured by Abbott was provided by a local supplier. Before any synthesis procedure was conducted, toluene and tetrahydrofuran (THF) were dried by reactive distillation with sodium. mPEG and PEG-diol were dried by azeotropic distillation with dried toluene. Traces of water in CL were removed using calcium hydride (CaH_2_) for 48 h and stored with a 3 Å molecular sieve. The other reagents were used without any additional purification.

### 2.2. Synthesis Procedures

#### 2.2.1. Synthesis of PEG-b-PCL Copolymers

In this study, ABCs of types AB and ABA composed of polycaprolactone (A) and polyethylene glycol (B) were synthesized by ring opening polymerization. Firstly, CL was polymerized by ring opening polymerization, starting from PEG as the initiator and Sn(Oct)_2_ as the catalyst. In typical synthesis conditions, mPEG (0.6 mmol) as the initiator and Sn(Oct)_2_ (0.6 mmol) as the catalyst are dissolved in dried toluene under Ar atmosphere, followed by CL (7.9 mmol). Here, the reaction mixture was maintained under stirring at 115 °C for 24 h. The ABA copolymer was synthesized in the same conditions but starting from PEG-diol as the initiator. The polymers were purified by passing them through a column packed with basic alumina and by three solvent precipitation cycles. The solid product was dried under reduced pressure at room temperature. In ^1^H-NMR (400 MHz, CDCl_3_, δ, ppm), the distinctive signals of PCL were as follows: 4.08 (t, J = 6.6), 2.33 (t, J = 6.6 Hz, 73H), 1.72–1.62 (m, 146H), 1.40 (m, 73H) and mPEG: 3.66 (s, 494H). The spectrum is shown in Figure S1A.

#### 2.2.2. Synthesis of PEG-PCL-COOH

AB and ABA copolymers were converted into their corresponding carboxylated materials by esterification with succinic anhydride following the steps of a previous procedure [39]. Briefly, the corresponding hydroxyl ended copolymer (5 mmol) and SA (6 mmol) were dissolved in 10 mL of chloroform. After adding DMAP (6 mmol) and TEA (6 mmol), the reaction mixture was maintained under stirring for 24 h in Ar atmosphere. In recovering the product, the reaction mixture was firstly filtrated, and then the modified polymer was precipitated by adding an excess of diethyl ether. The carboxylated copolymer was purified in two successive precipitations. Finally, the recovered solid was dried under vacuum at room temperature. In the ^1^H-NMR spectrum, apart from signals for the PEG and PCL segments, a new signal at 2.66 ppm due to methylene groups of succinic moiety also appeared. The spectrum is provided in Figure S1B.

#### 2.2.3. Synthesis of PEG-PCL-RET

Carboxylated copolymers were reacted with RET by the Steglich esterification procedure. The materials (2 mmol of –COOH) and DCC (1 mmol) were dissolved in 15 mL of DCM and stirred at 500 rpm for 30 min. Then, DMAP (6 mmol) and RET (5 mmol) were added. The reaction mixture was stirred at room temperature in darkness for 24 h [40]. Finally, the reaction mixture was filtered to remove the by-product *N*,*N*′-dicyclohexylurea. The RET-conjugated AB and ABA copolymers were purified by solvent precipitation with DCM and ethyl ether. Until the usage of these copolymers, they were stored at 6 °C under inert atmosphere.

### 2.3. Characterization Techniques

The ^1^H-NMR spectra of mPEG and HO-PEG-OH, AB and ABA copolymers and their corresponding carboxylated and RET-conjugated derivatives were recorded on a 400 Ultrashield spectrometer operated a 400 MHz (Bruker, Mannheim, Germany) by using CDCl3 as the solvent. The FTIR spectra were acquired in an IR Prestige 21 FTIR equipment (Shimadzu, Houston, TX, USA). The samples were dissolved in THF and deposited on NaCl window. After solvent evaporation, the resulting film was characterized by transmittance. Molecular weight distribution and dispersion index were determined by gel permeation chromatography using a high-performance liquid chromatographer (Waters, Pittsburgh, PA, USA) equipped with a differential refraction index detector. Analyses were performed in THF at a flow rate of 0.8 mL/min by using HR 4E column. The calibration curve was constructed with polystyrene standards in the range of 1–69 kDa.

The thermal behaviour of the polymeric materials was characterized by a differential scanning calorimeter (DSC, Model 822e, Mettler Toledo, Colombus, OH, USA) as follows. Firstly, thermal history was erased by heating from room temperature to 100 °C at 20 °C/min. Then, the samples were cooled down to −60 °C at 10 °C/min to allow for crystallization. Finally, the melting behaviour was determined by heating them to 250 °C at 10 °C/min.

The average size and distribution of particles were determined by dynamic light scattering (DLS, Litesizer 500, Anton Paar, Houston, TX, USA) operated at 27 °C and 37 °C. The samples were diluted with deionized water (approximately 18 MOhm-cm) to a concentration of 0.1%. 

### 2.4. Critical Micellar Concentration Measurements

The critical micellar concentration (CMC) of block copolymers was determined by fluorescence and using pyrene as a probe based on previously reported procedures [41,42,43]. Briefly, micellar dispersion of AB, ABA and their conjugates were prepared in various concentrations (0.01 to 1000 mg/L) with a fixed concentration of pyrene of 0.6 µM. The excitation spectra of pyrene from 300 to 360 nm were monitored at 390 nm for each dilution by using a Cary Eclipse fluoresce spectrometer (Agilent, Santa Clara, CA, USA). CMC was determined by plotting the ratio of the intensities at 335 and 332 nm (I_335_/I_332_) versos log concentrations.

### 2.5. Preparation of AmB Loaded Micelles

AmB@PMs was prepared in the modified nanoprecipitation method [43]. Briefly, 20-mg of the corresponding polymeric sample was completely dissolved in THF, and the resulting solution was dropped to 25.0 mL of deionized water at 25 °C. Simultaneously, 5 mg of AmB was dissolved in approximately 5 mL of methanol, and this solution was set to dropwise relative to the solution containing the polymer. The final mixture was maintained under continuous stirring for 48 h. After the complete evaporation of the solvent achieved under reduced pressure, non-encapsulated AmB was removed by centrifuging the medicated emulsions at 1.3 × 10^4^ RPM for 14 min. AmB@PM suspensions were dried by lyophilisation and maintained at −20 °C until the biological and physical characterizations were completed.

### 2.6. Determination of Encapsulated AmB

Encapsulated AmB was determined as follows. Approximately 1 mg of the corresponding formulation was dispersed in approximately 8 mL of methanol. This mixture was sonicated for 15 min at room temperature, and then the volume was completed to 10.00 mL. This mixture was centrifuged at 5000 RPM for 10 min to remove the polymer component. The supernatant was analysed by UV-vis at 406 nm. The measurement allowed for the determination of drug loading (%DL) and encapsulation efficiency (%EE) with Equations (1) and (2), respectively.

(1) $$\%DL=\frac{mass\;of\;encapsulated\;AmB}{mass\;of\;AmB@PMs}\;\times 100$$

(2) $$\%EE=\frac{mass\;of\;encapsulated\;AmB}{mass\;of\;feeded\;AmB}\times 100$$

### 2.7. Assessment of Aggregation State

The aggregation degree of AmB in the polymer micelles was studied by UV-vis spectrometry. The spectra of the solutions of AmB in DMSO and PBS, and the dispersions of AmB@PMs in PBS at pH 7.4, were directly acquired at 300–450 nm in an Evolution 300 UV/VIS spectrometer. All the solutions were prepared to a final concentration of AmB of 3.8 µg/mL.

### 2.8. Release Study

The release kinetics of AmB from formulated polymer micelles was assessed in vitro by employing the dialysis method [44]. A mass of each formulation corresponding to approximately 0.25 mg of AmB was dispersed in approximately 3 mL of PBS and then placed in a cylindrical tube capped with a dialysis membrane and MWCO of 10 KDa. The formulation was dialyzed against 40.0 mL of a solution of sodium deoxycholate (0.5%) and DMSO in the volume ratio of 2:1. The release was measured for 96 h at 37 °C. The profiles were obtained by periodically withdrawing aliquots of 1.00 mL. The measurement of the released AmB was performed by UV–vis.

The volume of the release medium was maintained constant by repositioning each drawn aliquot with fresh medium. The cumulative release (*Q*) was calculated as follows:

(3) $$Q=C_{n}V+\sum_{i=1}^{i=n-1}C_{i}V_{i},$$

where *V* is the volume of the release medium, and *C_n_* and *C_i_* correspond to concentration at a given time and the former aliquots with volume *V_i_*, respectively. 

### 2.9. In Vitro Biocompatibility

#### 2.9.1. Haemolysis

Analysis was performed following a published protocol [44]. Whole blood collected from O+ donors was added with an anticoagulant 1 mM of EDTA solution and then centrifuged at 500× *g* for 5 min to separate the RBCs. The recovered RBCs were rinsed twice with PBS and diluted in the same buffer to obtain an absorbance of 0.5 at 540 nm. Then, 190 µL of this cell dispersion was treated with 10 µL of a solution containing the corresponding testing material. PBS was used as a negative control, whereas in the presence of Triton X-100 complete haemolysis of RBCs is guaranteed. The treated solutions of RBCs were incubated at 37 °C under continuous shaking for 1 h. Afterward, the plaque was centrifuged at 500× *g* for 5 min to separate the non-lysed RBCs. The haemolysis was determined by measuring the absorbance of the haemoglobin in the solution at 541 nm as follows:

(4) $$Hemolysis\left(\%\right)=\frac{A_{s}-A_{NC}}{A_{PC}-A_{NC}}\times 100,$$

where *A_s_* is the absorbance of the drug-treated RBCs, and *A_NC_* and *A_PC_* are the absorbance values of the negative and positive controls, respectively.

#### 2.9.2. Cytotoxicity against Fibroblasts

The cytotoxicity of AmB@PMs was evaluated on mouse fibroblast (L929) using the MTT colorimetric assay following a previously published protocol [44]. Briefly, fibroblasts were seeded at a density of 2.1 × 10^4^ cells/well, allowed to adhere and proliferate for 24 h in DMEN medium. Wells containing the adhered cells were treated with dispersions of AmB@PMs corresponding to concentrations of AmB of 0.05 to 15 mg/L. The cell cultures were incubated along 24 h at 37 °C under a 5% CO_2_ atmosphere. Finally, the medium was replaced with 30 µL of a solution of 1 mg/L of MTT in PBS. The treated cells were incubated at 37 °C for 4 h, then MTT was removed. The resulting Formazan crystals were solubilized with 100 µL, and analysed in an ELx800 reader (BioTek, Winooski, VT, USA) at 490 nm. The absorbance values were computed as indicated in Equation (5) to determine cell viabilities.

(5) $$Cell\;Viability\;\left(\%\right)=\frac{A_{Sample}}{A_{Blank}}\times 100,$$

where *A_sample_* is the absorbance of AmB@PMs and Fungizone^®^ treated well, and *A_Blank_* is the absorbance of control well without drug treatment.

### 2.10. In Vitro Assessment of Antifungal Activity

An antifungal susceptibility test was carried in accordance with Clinical and Laboratory Standards Institute’s (CLSI) broth microdilution method (BMD) following the M27-A3 guidelines [45,46]. Dilutions of AmB@PMs and Fungizone^®^ with concentrations of AmB in the range of 0.11–7.5 µg/mL were evaluated. Minimum Inhibitory Concentrations (MICs) were determined visually after 24 h as the lowest concentration of drug that can cause considerable diminution relative to the drug-free growth control. The MICs were determined visually and supported by measurements of absorbance, which was determined by spectrophotometry at 600 nm. The values obtained at this phase were further corroborated employing resazurin (7 mM) as a redox indicator. Resazurin is a blue non-toxic cell-permeable compound and virtually non-fluorescent. Upon entering living cells, resazurin is reduced to resorufin, a compound that is violet to pink and highly fluorescent [47,48].

## 3. Results

### 3.1. Synthesis of Polymeric Precursors

**Scheme 1:**
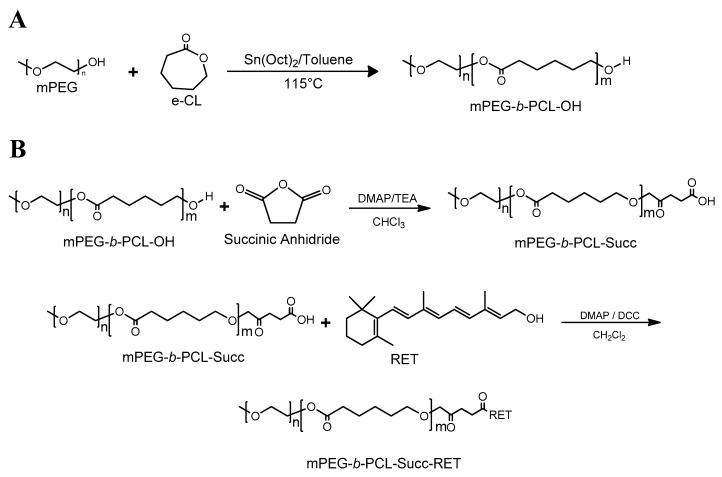
Synthesis pathway. (**A**) Ring Opening Polymerization (ROP) of CL by using mPEG as the initiator, and (**B**) conjugation of mPEG-b-PCL with RET by Steglich esterification.

mPEG-b-PCL (AB) and PLC-b-PEG-b-PCL (ABA) block copolymers were synthesized by Ring Opening Polymerization (ROP) starting from PEG as the macroinitiator (Scheme 1A). Characterizations by ^1^H-NMR confirmed that the proposed structure was achieved. The average molecular weights (Mn) are listed in Table 1. The dispersion indexes estimated by gel permeation chromatography coincide with values reported for polymerization catalysed by Sn(Oct)_2_ [49]. 

**Figure 1:**
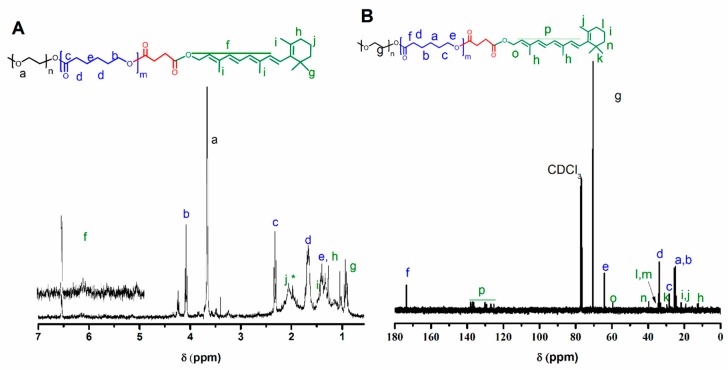
(**A**) ^1^H and (**B**) ^13^C-NMR spectra of mPEG-b-PCL conjugated with retinol and their corresponding signal designation.

Afterward, the resulting AB and ABA copolymers were reacted with succinic anhydride to obtain a carboxylate copolymer at the PCL ends. The occurrence of this reaction was confirmed by the appearance of a new signal near 2.7 ppm due to succinic acid end moieties (Figure S1). The carboxylated materials were subsequently esterified with RET by the Steglich process in the presence of DCC and DMAP as the coupling agent and the catalyst, respectively [50] (Scheme 1B). The occurrence of this reaction was confirmed by ^1^H-NMR and ^13^C-NMR. The corresponding spectra of the isolated products (Figure 1A,B) have signals resulting from PEG, PCL and RET moieties. 2D-NMR spectra were not feasible given the low number of RET nuclei compared to the copolymer.

### 3.2. Critical Micellar Concentration

In aqueous media, ABCs, as a result of solvophobic forces, assemble themselves to produce aggregates; among them, micelles are more frequently reported for low molecular weight copolymers [30]. This kind of core–shell structure is spontaneously formed when the concentration of ABC is larger than its CMC. Thus, determining the CMC allows for the characterization of the thermodynamic favourability of the self-assembling process, as well as the stability of the resulting nanostructures against dilution. Additional to thermodynamic stability, PMs even at concentrations below the CMC of the corresponding ABC remain stable for long periods as a result of intermolecular interaction in polymer segments [51]. Conversely, in the presence of a drug the stability of the PMs (not studied here) is altered depending on the nature of the polymer–cargo interactions [52].

**Figure 2:**
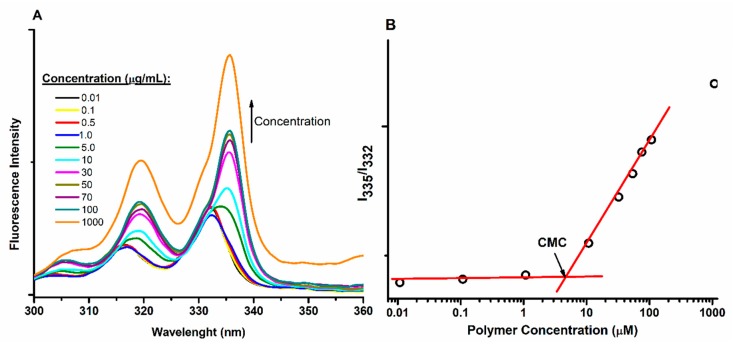
CMC measurement by fluorescence employing pyrene as the probe molecule. (**A**) Emission spectra of solutions containing different concentration of a representative sample. (**B**) Plot of I_335_/I_332_ used to measure CMC values.

In this study, CMC values were measured by fluorimetry employing pyrene as a probe molecule, this technique apart from being a simple method, has also shown to be sensitive to obtain reliable values [43,53,54]. Figure 2A shows a spectral comparison of pyrene dissolved in solutions with different concentration of representative copolymers. Apart from the obvious increase in fluorescence intensity when the concentration increases, a red shift of the maxima is also observed. Figure 2B presents a plot of the ratio of intensities at 335 and 332 nm. At the low copolymer concentration, this ratio is kept constant. However, the ratio increases at certain concentration. This phenomenon reveals the presence of micelles and that CMC has been achieved, thus allowing for the measurement of the values listed in Table 1. According to these results, the AB copolymer exhibits a larger CMC than ABA, suggesting a more hydrophilic character. In conjugation with RET, the CMC increases, showing a tendency opposed to the highly hydrophobic character of the PCL segment that was achieved in the presence of RET. This finding suggests that the conjugation of AB and ABA decreases segmental flexibility, corresponding to a relatively high steric hindrance that increase of energy required to achieve conformational changes required for the micelles formations.

### 3.3. Differential Scanning Calorimetry

DSC was used to study the effect of the conjugation of AB and ABA copolymers with RET on their thermal properties (i.e., their morphology). After erasing the thermal history of the samples, the crystallization of the samples was studied by cooling them down from 100 to −20 °C at 10 °C/min, while heating the samples to as high 100 °C at 10 °C/min allowed for the characterization of their melting. 

**Figure 3:**
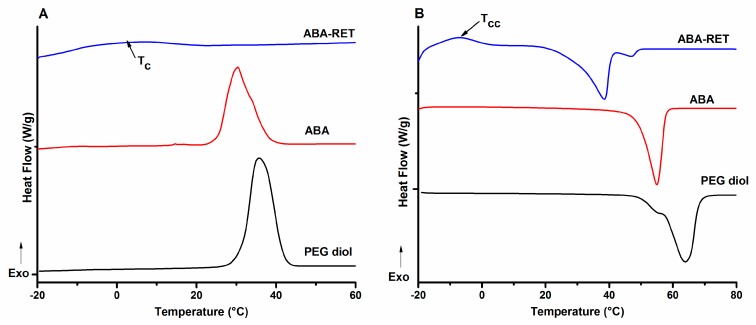
DSC traces for a representative set of materials: PEG-diol, PLC-b-PEG-b-PCL (ABA and its corresponding conjugate ABA-RET). Cooling (**A**) and heating (**B**).

According to Figure 3A showing representative traces of ABA, PEG-diol and ABA-RET, the samples exhibit a crystallization transition characterized by the release of thermal energy corresponding to crystallization enthalpy. Table 2 lists the temperatures that correspond to the maxima of the crystallization peaks. According to these values, PEG homopolymers crystallize at a higher temperature than the corresponding copolymers, proving that CH_2_−O−CH_2_ linkages endow PEG flexibility and allows for the chain folding required to achieve high crystallization. Upon copolymerization with PCL to obtain AB and ABA copolymers, the crystallization temperature is shifted to a low temperature, indicating that this transition is kinetically less favoured. Cooling traces of AB support the occurrence of two separated crystallization phenomena, while ABA exhibited a single peak with a shoulder at the high temperature corresponding to the superposition of two transitions; these findings suggest the formation of segregated crystalline domains [55,56]. Moreover, in conjugation with RET, the crystallization was delayed, as deduced from the shift to the low temperature, and the fact that transition became less pronounced, as seen for ABA-RET in Figure 3B. The presence of RET reduced the crystallinity of both segments by reducing their segmental mobility.

Figure 3B shows the DSC traces obtained in the heating stage for the representative materials. PEG-diol exhibits two melting peaks characterizing different crystalline forms of PEG [57]. However, ABA only presents a broad peak with a maximum at 54.4 °C that can be attributed to the melting of both PEG and PCL domains. The depletion of T_m_ of the mPEG segment agrees with a low intense crystallization peak, low crystallization degree and a decrease of size of crystalline domains. When ABA was conjugated with RET, besides the notable decrease in T_m_, the appearance of a cool crystallization (T_cc_) peak at approximately −10 °C corroborates that RET hinders the crystallization of both PEG and PCL domains. The data shown in Table 2 indicate the same tendency for the diblock materials. Meanwhile, melting enthalpy (ΔH_m_), which was estimated by the integration of melting peaks, corroborates the decrease in crystallization degree upon the conjugation with RET; this phenomenon is in concordance with the reduction of segmental mobility.

### 3.4. AmB Encapsulation

The encapsulation of AmB in polymer micelles was carried out by a partition method. A colloidal dispersion of the preformed micelles was added with AmB dissolved in an excess of methanol. This setup allowed for the diffusion of AmB from the continuous medium to the micelle core. When methanol evaporates, AmB can self-assemble in an aqueous medium to produce insoluble aggregates, or it can interact with the components of micelles driven to its encapsulation. Table 3 contains the results of the quantification of encapsulated AmB carried out on the lyophilized formulations after extracting the AmB with methanol. The analysis of its concentration was performed with UV-vis spectroscopy to obtain the provided DL and EE values.

The results indicate that encapsulation efficiency and loading capacity increase in the presence of RET, suggesting favourable polymer–AmB chemical interactions as corroborated by the most pronounced increase achieved for ABA-RET. Both RET and AmB presented a polyenic segment that likely favours π–π interactions [39]. This tendency agrees well with previous findings that corroborate an increase in encapsulation efficiency when PEG-b-PCL copolymers are conjugated with cholesterol [44]. Similarly, the conjugation of AB and ABA copolymers with RET decreases their crystallinity, thereby increasing the free volume of the polymer chains, which in turn favours the encapsulation of the drugs.

Both empty micelles and micelles loaded with AmB were characterized by dynamic light scattering at 25 °C and 37 °C to determine their sizes and distributions (Table 4). Empty PMs composed of RET-conjugated copolymers presented a larger diameter than the corresponding initial material, indicating that the structure of the micelles and the nature of the interactions leading to their formation depend on the structure of the copolymers. A3s the temperature is increased, the diameter of the particles also increases, suggesting a reduction of the solvation of the hydrophilic PEG segment. Thus, the aggregation of particles is enabled [58]. Likewise, a temperature augment could also cause a larger aggregation number as well as a change of the polymer chains conformations [59,60]. According to DLS results, no evidence about AmB leakage upon heating the colloidal dispersion was obtained.

**Figure 4:**
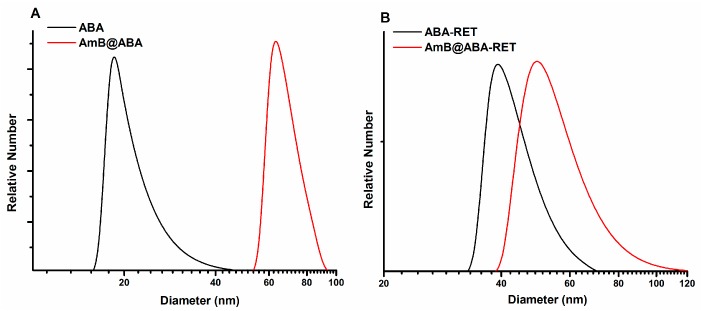
Diameter distributions of empty micelles and AmB@PMS obtained from ABA (**A**) and ABA-RET (**B**) copolymers.

Figure 4 shows a comparison of the distribution plots of empty ABA and ABA-RET with their corresponding formulations at 37 °C. In the presence of AmB, the diameter of the nanoparticles increased markedly, suggesting that AmB is not only dissolved in the hydrophobic micelle nucleus but is also involved in particle formations, which we assume is favoured by the hydrophobic AmB–polymer interactions. Transmission electron microscopy images provided in Figure S2, indicate that AmB loaded micelles are spherical with diameters below 100 nm.

### 3.5. Assessment of AmB Aggregation

AmB, given its amphiphilic character, tends to aggregate when its concentration exceeds CAC. According to Zielińska et al. [61], the aggregation proceeds via hydrophobic interactions, while the interactions between polar groups and their amphoteric character have minimal contribution. Assessing the aggregation state of AmB in antifungal formulations allows for an estimation of its toxicity and effectivity. The toxicity of AmB is mainly adjudicated to water-soluble aggregates (including dimers), whereas insoluble poly-aggregates have been reported to be safer [62]. Monomeric AmB that accounts for a high antifungal action, presents a minimal toxicity compared with the soluble aggregates [25].

**Figure 5:**
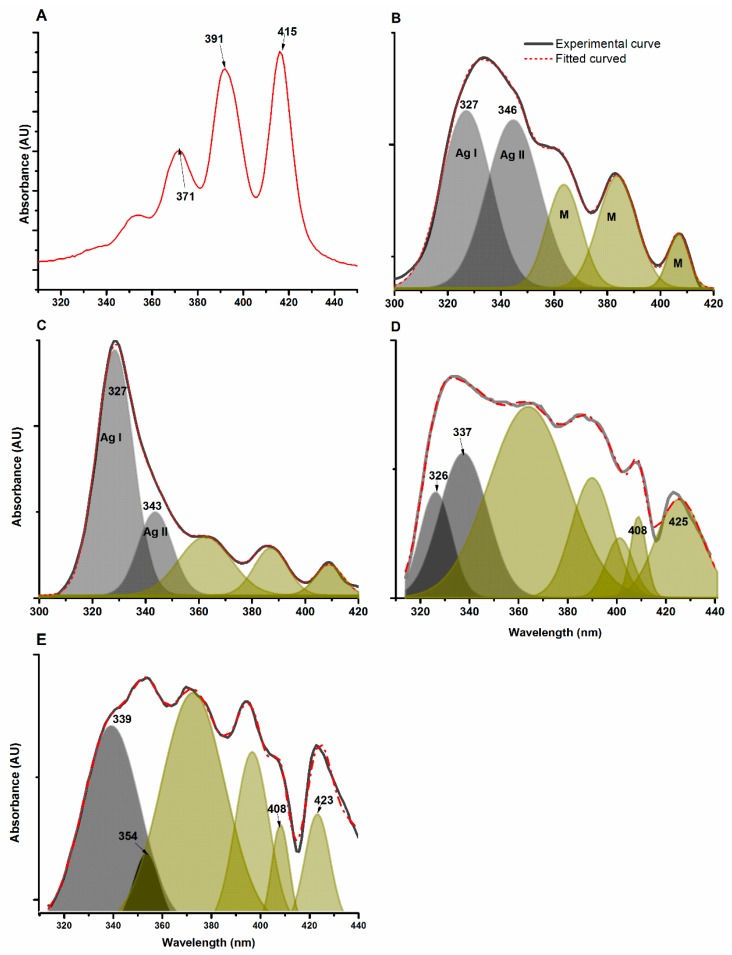
UV–vis spectrum of AmB dissolved in PBS (**A**) in water (**B**), Fungizone^®^ (**C**), AmB@ABA (**D**) and AmB@ABA-RET (**E**). The spectra were fitted to Gaussian peaks to allow for the separation of absorption peaks due to two types of aggregates, designated as AI and AII, and the monomeric form (M).

UV-vis spectroscopy is a reliable technique to characterize the aggregation state of AmB because its spectral features depend on the molecular arrangements obtained by its self-aggregation in the presence of surfactants, solvents and biomolecules. The spectrum of AmB dissolved in DMSO corresponding to the monomeric state [63] exhibited λ_max_ at 415, 391, and 371 nm (Figure 5A). Conversely, in the spectrum of aqueous AmB, absorption maxima at 408, 385, and 365 nm are principally due to the monomeric form of the drug [64]. The absorption band at 345 nm is indicative of its aggregated form (Figure 5B). Similar tendencies are observed in the spectrum of Fungizone^®^ (Figure 5C) and those of AmB@PMs obtained from ABA and ABA-RET (Figure 5D,E).

The AmB spectra were further processed by fitting them to Gaussian peaks as a means to estimate the relative amount of each type of arrangement. Figure 5B shows the spectrum of AmB in PBS and the corresponding fitted spectrum. In this case, five Gaussian peaks were necessary to fit the experimental data. The peaks at 327 and 346 nm correspond to different aggregates of AmB (AI and AII) with different molecular arrangements of AmB and intermolecular distances. The contributions of the monomeric form on the absorption spectra are also observed. The spectra of Fungizone^®^ have a similar absorption given the same wavelength observed for AmB dissolved in PBS, suggesting similar arrangements of AmB in the aggregates and in the monomeric form.

As for the encapsulation of AmB in PM, the absorptions were dependent on the composition of the copolymer. In the ABA, the aggregates presented absorption at the same λ_max_ of AmB in PBS and Fungizone^®^. In the presence of RET, the absorption of this fraction of AmB is shifted to a relatively high λ, suggesting an increase in distance between AmB molecules involved in the formation of aggregates, and thus less-dense structures are obtained [65]. In both formulations, the absorption at the longest wavelength corresponding to monomeric species gave rise to tow absorption bands with λ_max_ at 423 and 408 nm. The bathochromic absorption suggests that a significant fraction of AmB underwent an increase in hydrophobicity around the heptaene moieties [22]; this finding provides evidence on the nature of the drug–polymer interaction that drive its encapsulation.

In UV–vis spectrometry, absorbance in a given λ is proportional to the concentration of the absorbing compound. Here, the fitting peaks are integrated to let for the obtainment of the data provided in Table 5. The relative area of the peaks with relatively large λ is proportional to the fraction of aggregated AmB. These values indicate that AmB, in its encapsulation for on PMs, is mostly in its monomeric form. By contrast, AmB dissolved in PBS and Fungizone^®^ is mostly aggregated, and the minor fraction of monomers corresponds to CAC. Additionally, the relative amount of each type of aggregate is evaluated by determining the relative area of the aggregated AI type. The relative amounts are similar for both types of aggregates in AmB dissolved in PBS; however, the aggregates in Fungizone^®^ with closely packed AmB molecules (AI) are the predominant arrangements. These results agree with the blue-shifting absorption associated with the aggregates previously reported for AmB in Fungizone^®^ formulations [65,66]. 

In the case of micellar formulations, the aggregate distribution depends on the copolymer structures. For ABA, the aggregate type AII is the predominant arrangement, suggesting that the hydrophobic segments of PCL can reduce the formation of tight aggregates, probably by reducing the mobility of AmB molecules dissolved in the core of micelles. In the presence of RET, aggregates type AI is not detected; instead, the weak absorption at 354 nm suggests the formation of a new type of molecular arrangement that presumably involves RET moieties.

### 3.6. Release Study

**Figure 6:**
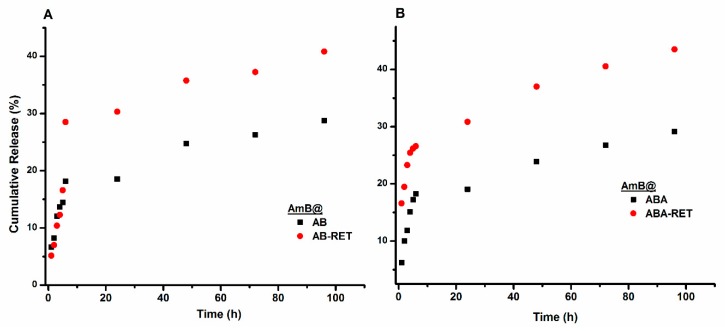
Release profiles of AmB loaded in AB (**A**) and ABA (**B**) and their corresponding conjugates with RET.

The release of AmB from micellar formulations was assessed at 37 °C by dialysis and by using a solution of sodium deoxycholate and DMSO as the release medium to guarantee sink conditions (AmB solubility > 20 µg/mL). The concentration of AmB released to the medium was monitored using UV-vis spectroscopy at a wavelength of 406 nm. The release profiles are shown in Figure 6. The curves characterize two different behaviours. During the first 6 h, AmB is quickly released to obtain a cumulative release close to 30%. After 6 h, the release rate decreased, but the formulations continued to release the drug at a near-constant rate.

In determining the effect of RET presence on release behaviour, the profiles of representative formulations of AmB@ABA and AmB@ABA-RET were analysed on the basis of the models described in Table S1. The data corresponding to each step shown in the release profiles in Figure 6B are independently fitted to the equation representing each model. The derived values are listed in Table 6. For the initial stage (first 6 h), the release profiles fitted well with the Korsmeyer–Peppas model [67,68]. Meanwhile, AmB@ABA obtained an *n* value of 0.68 for the supposed spherical particles, indicating an anomalous behaviour. In other words, AmB can be released by both diffusion and swelling mechanisms. The value of 0.283 obtained for AmB@ABA-RET agrees well with the diffusion or Fickian mechanism. The same trend for the second stage can be deduced from the fitting parameters. Both formulations exhibited relatively low k values that agree with the reduction of the releasing rate at prolonged times. The value of this parameter in both stages also suggest that the conjugation with RET facilities the release of AmB, presumably due to the relatively low crystallinity and thus less-dense micelle nuclei. 

In vivo, the release of AmB is facilitated by the interaction of the drug and nanocarriers with plasmatic constituents such as lipoproteins. Although in vitro those conditions cannot be emulated, a release medium that provides sink conditions allows for comparing the ability of nanostructured vehicles to control the release of AmB.

### 3.7. In Vitro Biocompatibility

**Figure 7:**
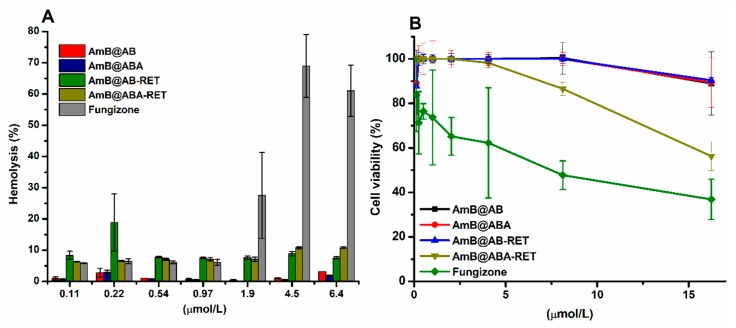
(**A**) Haemolytic profiles of AmB@PMs and Fungizone^®^ and (**B**) Cell viability of mouse fibroblasts (L929) in the presence of AmB formulations.

The haemolytic behaviour of AmB@PMs was compared with that of Fungizone^®^. RBCs were incubated with different amounts of each formulation. The haemolysis extent is plotted in Figure 7A. As previously discussed, the toxicity of AmB against mammalian cells, such as RBCs, is attributed to the formation of aggregates. This phenomenon results in the complexation of cholesterol in cell membranes and subsequently leads to the formation of pores, which then serve as leaking points of essential substances. Fungizone^®^ is a colloidal dispersion of AmB stabilized by sodium deoxycholate (i.e., used to improve its solubility). In concordance with its high aggregation deduced from the UV-vis analysis, this formulation presented relatively high haemolytic activity, which is more pronounced at the concentrations superior to CAC of AmB (1 µmol/L). By contrast, when AmB is encapsulated in polymer micelles (i.e., to provide controlled release and avoid aggregation), its cytotoxicity is satisfactorily reduced in all concentrations. Micellar formulations with RET-conjugated precursors are more haemolytic than the corresponding AB and ABA precursors. Figure S3 shows a plot of the haemolysis extent for the micellar formulations and Fungizone^®^ as a function of their aggregation ratios (Table 5) with concentrations of approximately 4.2 µmol/L. As the aggregation of AmB increases, their haemolytic activity also increases, indicating that reducing the aggregation of AmB lets for safe formulations.

The cytotoxicity of AmB@PMs was further studied against fibroblast L929, and compared with Fungizone^®^. According to Figure 7B, the micellar formulations elicited lower toxicity. At the highest concentrations of AmB of 16 µmol/L, while Fungizone^®^-treated cells presented a viability lower than 50%, the cytotoxicity of the micellar formulations was lower than 30%. As formerly discussed, one of the main concerns in the administration of AmB is its renal toxicity, which is related to the ability of this drug to interact with cholesterol and induce the formation of pores in mammal cell membranes. A decrease of haemolytic activity suggests that the developed PMs attenuated the intrinsic toxicity of AmB, thereby a reduction of the in vivo nephrotoxicity is expected [69]. Likewise, fibroblasts L929 proliferation assays are in concordance with biocompatible formulations [70].

### 3.8. In Vitro Effectiveness

The efficacies of the micellar formulations of AmB and Fungizone^®^ were assessed against a set of reference ATCC and clinical strains, as listed in Table 7, along with their susceptibility profile determined according to CLSI [68]. In this group, an isolate resistant to conventional antifungals of parenteral administration was also included. In all cases, MIC was taken as the minimum concentration of AmB for the inhibited growth of yeast, the values were determined visually and confirmed employing resazurin. This violet substance is transformed to pink upon entering living cells, enabling to differentiate between alive and dead fungal cells, as shown in Figure S4 [47,48]. The derived results indicate that the broad spectrum of action of AmB can be maintained. In the case of *Candida auris* 537 resistant to AmB, the formulation of AmB@ABA inhibits its growing at 0.93 µg/mL concentration compared with the 3.75 µg/mL concentration for Fungizone^®^. Moreover, this result was further confirmed by the growing plots depicted in Figure S5. In most cases, micellar formulations exhibit higher efficacy than Fungizone^®^ against the evaluated strains, as deduced from the measured MIC values.

## 4. Conclusions

Copolymers composed of PEG and PCL are promising as micellar vehicles for drug delivery because of their biocompatibility. However, the absence of functional groups on PCL hinders their interaction with drugs which in turn results in low loading capacities. It was demonstrated that upon the conjugation of these block copolymers with RET, the encapsulation of AmB was enhanced to achieve values comparable with commercial liposomal formulations such as AmBisome, Lambin and Lipholyn and superior to Fungisome [12]. Upon its encapsulation, AmB retained its antifungal spectrum and presented an attenuated toxicity. Moreover, these nanocarriers improved the antifungal activity of AmB against most of the tested yeast strains as deduced from a reduction of the MICs of up to eight-fold compared with Fungizone^®^. Both, a high loading capacity and an improved efficacy are of paramount importance for the translation of new formulations to clinical practice, because they allow for more favourable cost-benefit ratios, and also a reduction of the toxicity risks associated to the polymeric vehicles and their metabolic products.

## Figures and Tables

**Table 1:** Characterization of mPEG-b-PCL (AB) and PLC-b-PEG-b-PCL (ABA) and their corresponding conjugated structures.

Sample	Composition ^a^	M*_n_* (kDa) ^a^	M*_w_*/M*_n_* ^b^	CMC	
				mg/L	μM
AB	(mPEG)_114_-(PCL)_11_-OH	6.27	1.20	4.5 ± 0.26	4.9
AB-RET	(mPEG)_114_-b-(PCL)_11_-RET	6.56	1.24	7.4 ± 0.78	8.0
ABA	HO-(PCL)_9_-(PEG)_136_-(PCL)_9_-OH	8.04	1.13	2.7 ± 0.86	2.9
ABA-RET	RET-(PCL)_9_-(PEG)_136_-(PCL)_9_-RET	8.61	1.29	5.5 ± 0.72	5.9

^a^ Determined by integration of ^1^H-NMR signals at 3.66 pm assigned to methylene groups of PEG which was taken as a molecular weight reference and –CH_2_O groups of PCL at 4.08 ppm, and ^b^ measured by GPC by employing a relative calibration plot.

**Table 2:** Thermal properties of the polymeric materials measured by differential scanning calorimeter (DSC).

Sample	Thermal Properties		
	T_c_ (°C)	T_m_ (°C)	ΔH_m_ (J/g)
mPEG	40.6	60.2	171.9
AB	28.2 18.6	48.0	86.2
AB-RET	23.3 −7.15	39.4	61.9
PEG-diol	35.4	63.9	107.9
ABA	30.8	54.4	110.7
ABA-RET	2.7	38.41 49.1	41.8

**Table 3:** Drug loading (DL) and encapsulation efficiency (EE) obtained by analysis by UV-vis the lyophilized formulations.

Formulation	EE (%)	DL (%)
AmB@AB	13.7 ± 0.71	2.74 ± 0.17
AmB@AB-RET	51.05 ± 0.79	10.21 ± 0.21
AmB@ABA	10.19 ± 0.12	2.38 ± 0.03
AmB@ABA-RET	33.65 ± 1.23	6.73 ± 0.28

**Table 4:** Average diameters measured by dyanimc light scattering (DLS) for empty micelles and AmB@PMs.

Formulation	Empty Micelles				AmB@PMs			
	25 °C		37 °C		25 °C		37 °C	
	Dh (nm)	PDI	Dh (nm)	PDI	Dh (nm)	PDI	Dh (nm)	PDI
AmB@AB	31.1 ± 1.7	0.172 ± 0.032	35.5 ± 2.8	0.189 ± 0.081	57.7 ± 4.3	0.324 ± 0.072	69.0 ± 6.0	0.234 ± 0.032
AmB@AB-RET	57.5 ± 4.2	0.213 ± 0.023	80.5 ± 7.5	0.204 ± 0.035	91.0 ± 3.5	0.391 ± 0.058	115.3 ± 7.2	0.359 ± 0.043
AmB@ABA	22.0 ± 2.3	0.198 ± 0.035	32.4 ± 3.3	0.212 ± 0.014	45.9 ± 4.7	0.429 ± 0.063	67.8 ± 3.5	0.354 ± 0.065
AmB@ABA-RET	53.4 ± 7.3	0.321 ± 0.041	77.6 ± 9.1	0.271 ± 0.037	80.2 ± 5.9	0.407 ± 0.024	81.7 ± 5.2	0.428 ± 0.027

**Table 5:** Summary of UV-vis spectrum analysis.

Sample	Aggregation Ratio *	AgI/Ag **
AmB in PBS	0.66	0.49
Fungizone^®^	0.70	0.74
AmB@AB	0.21	0.17
AmB@AB-RET	0.32	0.75
AmB@ABA	0.23	0.14
AmB@ABA-RET	0.37	0.81

* Estimated as the ratio of the area of the absorption due to the aggregates to the total area. ** Ratio of area of the absorption of AI to the total area of the absorptions due to the aggregates.

**Table 6:** Kinetics analysis for the release models.

Model	Parameter	ABA	ABA-RET	ABA	ABA-RET
**Zero order**	R^2^	0.975	0.903	0.973	0.969
	K (%·h^−1^)	2.426	2.061	0.138	0.173
**First order**	R^2^	0.917	0.878	0.950	0.950
	k (h^−1^)	0.207	0.095	0.006	0.005
**Higuchi**	R^2^	0.992	0.955	0.996	0.994
	k (%·h^−0.5^)	0.085	0.074	0.021	0.026
**Korsmeyer–Peppas**	R^2^	0.994	0.975	0.999	0.999
	n	0.608	0.283	0.613	0.496
	k (h^−n^)	0.063	0.166	0.072	0.141

**Table 7:** Values of minimum inhibitory concentration (MIC) of AmB@PMs formulations compare with Fungizone^®^ and profiles of susceptibilities of *Candida spp.* to fluconazol and caspofungine by CLSI reference methodology.

Yeast	Reference	CLSI			Formulations			
		FLC *	CAS *	Fungizone^®^	AmB@AB	AmB@ABA	AmB@AB-RET	AmB@ABA-RET
		MIC (µg/mL)						
*C. albicans*	SC5314	1	0.06	0.46	<0.11	<0.11	<0.11	<0.11
*C. glabrata*	ATCC 2001	0.25	0.06	0.46	<0.11	<0.11	<0.11	<0.11
*C. krusei*	ATCC 6258	32	0.25	1.875	0.23	0.23	0.43	0.23
*C. parapsilosis*	ATCC 22019	1	0.25	0.23	<0.11	<0.11	<0.11	<0.11
*C. auris*	435-PUJ-HUSI	8	0.25	0.93	0.23	0.23	0.46	0.23
C. auris *	537-PUJ-HUSI	128 **	<0.05	3.75	1.865	0.93	3.75	3.75
*C. parapsilosis*	75-PUJ-FVL	0.125	<0.05	0.11	<0.11	<0.11	0.11	<0.11

* FLC: Fluconazole*CAS: caspofungin. ** This MIC corresponds to a resistance isolate.

## Supplementary Materials

The following are available online at https://www.mdpi.com/1999-4923/12/3/196/s1, Figure S1. 1H-NMR spectra of A. ABA and B. ABA-Succinic acid copolymers. Figure S2. TEM image of micellar dispersion of sample AmB@ABA-RET showing the presence of spherical nanoparticles. Figure S3. Plot of relative haemolysis of each formulation at the concentration of 3.8 µg/mL versus its corresponding aggregation ratio value listed in Table 5, Figure S4. Determination of MIC employing resazurin. Figure S5. Growing curves of *C. auris* 537 in the presence of different concentrations of AmB@PMs, Table S1. Release models.
