# Supplementary material for: Development of Amphotericin B Micellar Formulations Based on Copolymers of Poly(ethylene glycol) and Poly(ε-caprolactone) Conjugated with Retinol
Source: Pharmaceutics. 2020 Feb 25;12(3):196. doi: 10.3390/pharmaceutics12030196 (PMC7150995; doi:10.3390/pharmaceutics12030196)
Supplement: Supplementary file 1 [file pharmaceutics-12-00196-s001.pdf]

Article

Supplementary Materials: Development of Amphotericin B micellar formulations based on copolymers of poly(ethylene glycol) and poly(ε-caprolactone) conjugated with retinol

Yeimy J. Rodriguez, Luis F. Quejada, Jean C. Villamil, Yolima Baena, Claudia M. Parra-Giraldo and Leon D. Perez.

Table S1. Release models [1].

Model	Equation	Features
**Order zero**	$Q_t = Q_0 + k_0 t$	The release is given as a function of time at a constant rate, independent of the concentration of the drug.
**Order one**	$LnQ_t = LnQ_0 - k_1 t$	The released amount is proportional to the quantity of drug that remains in the matrix system; therefore, it tends to decrease as a function of time
**Higuchi**	$Q_t = k_H t^{1/2}$	The release is directly proportional to the square root of time
**Korsmeyer-Peppas**	$\frac{M_t}{M_\infty} = k_{kp} t^n$	Depending on the value of n it is possible to establish the release mechanism (for a sphere):<br>Fickian diffusion: $n = 0.43$<br>Anomalous transport: $0.43 < n < 0.85$<br>Order zero: $n = 1.0$<br>Transport type II: $n > 0.85$

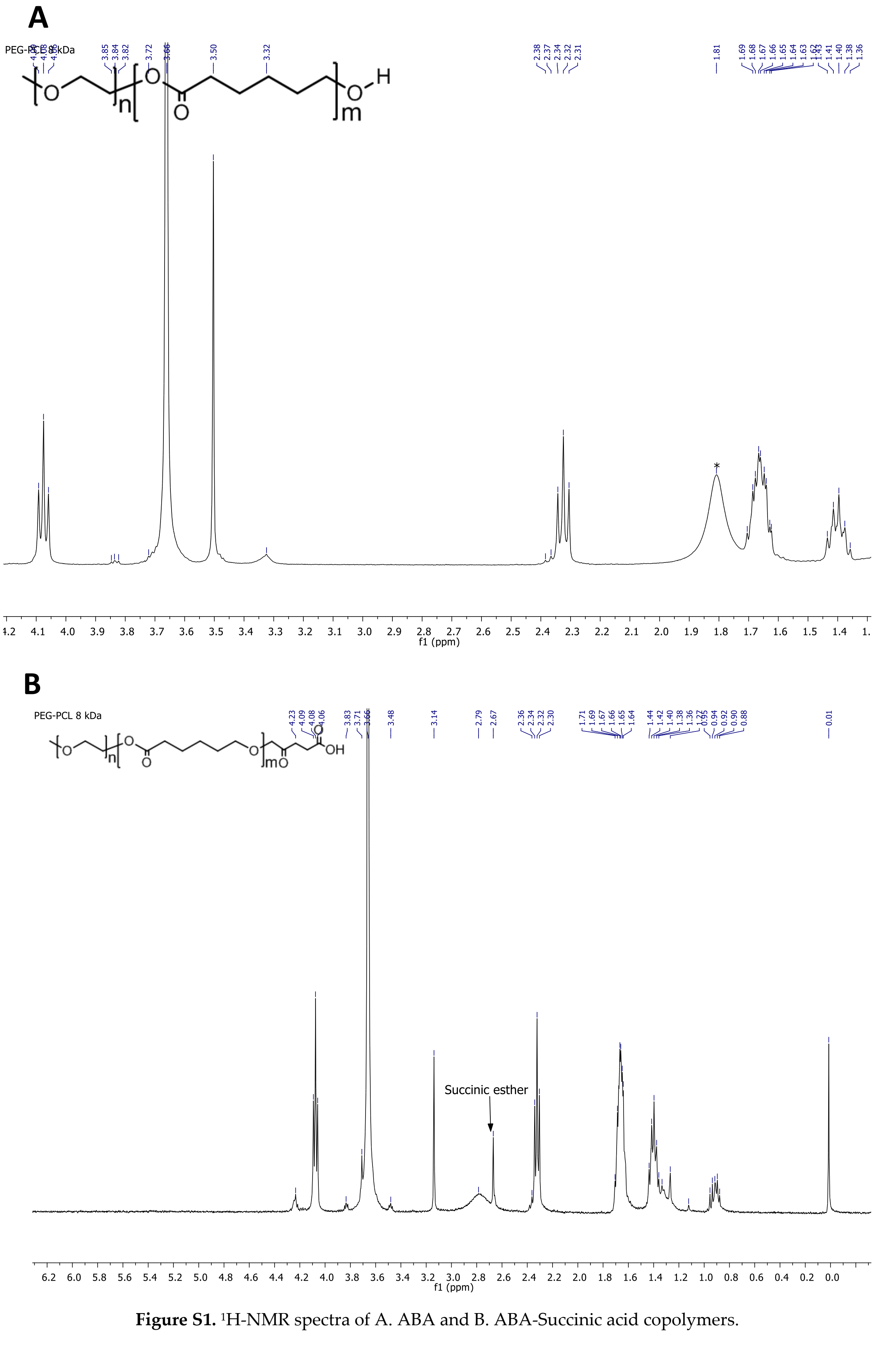

Figure S1. ^{1}H-NMR spectra of A. ABA and B. ABA-Succinic acid copolymers.

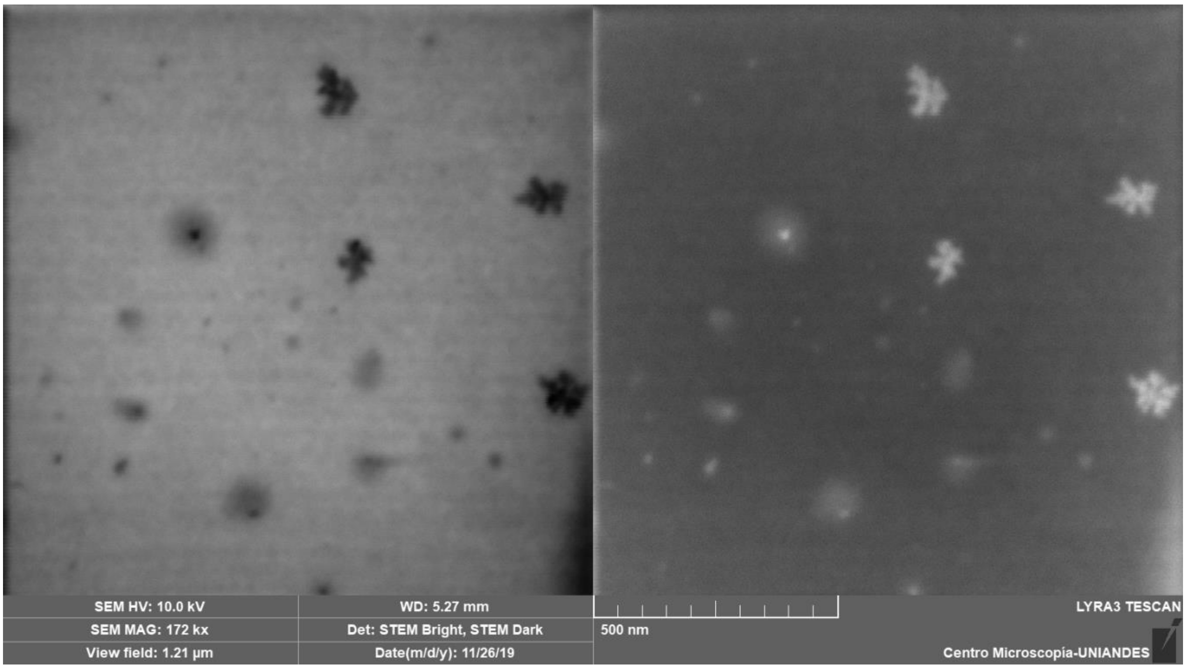

Figure S2. TEM image of micellar dispersion of sample AmB@ABA-RET showing the presence of spherical nanoparticles.

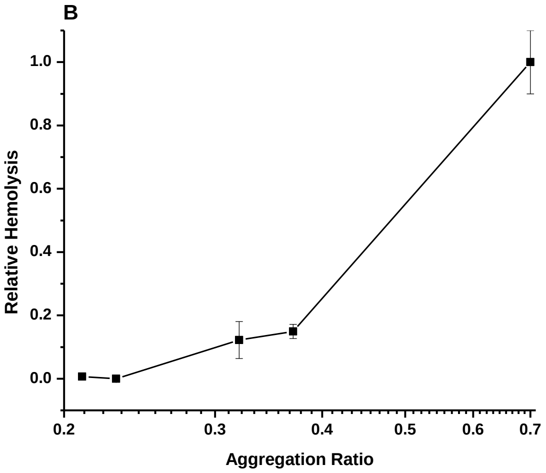

Figure S3. Plot of relative haemolysis of each formulation at the concentration of 3.8 μg/mL versus its corresponding aggregation ratio value listed in Table 5.

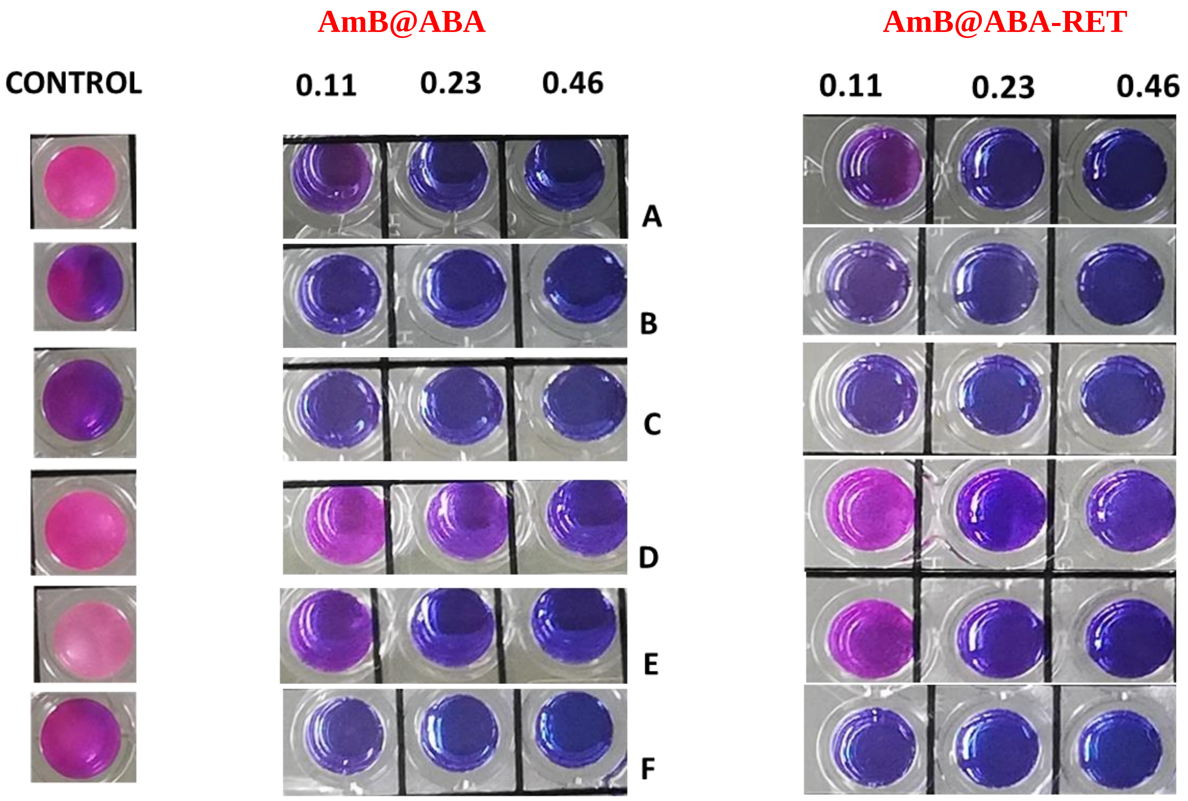

Figure S4. Determination of MIC employing resazurin. In the wells: (**A**) C. krusei ATCC 6258; (**B**) C. parapsilosis ATCC 22019; (**C**) C. glabrata ATCC 2001 (D) C. auris HUSI 435; (E) C. tropicalis ATCC 1018; (F) C. parapsilosis HUSI 75

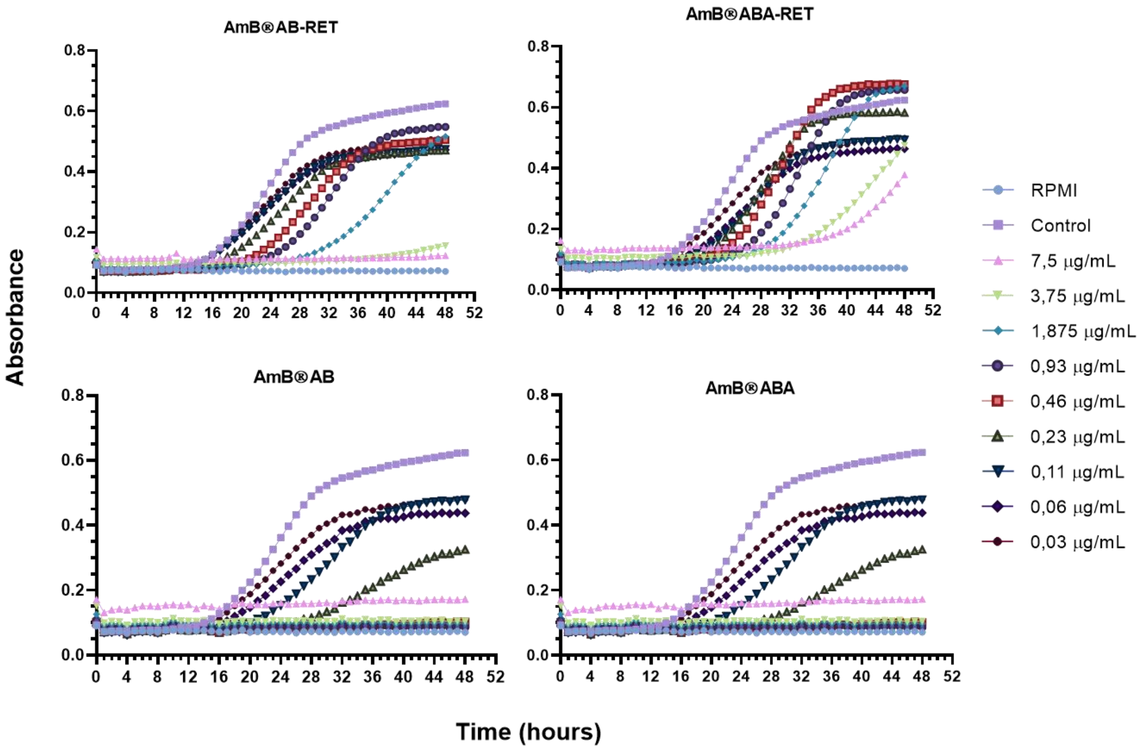

Figure S5. Growing curves of C. auris 537 in the presence of different concentrations of AmB@PMs.

References

1. Barzegar-Jalali, M.; Adibkia, K.; Valizadeh, H.; Shadbad, M. R. S.; Nokhodchi, A.; Omidi, Y.; Mohammadi, G.; Nezhadi, S. H.; Hasan, M., Kinetic analysis of drug release from nanoparticles. *Journal of Pharmacy and Pharmaceutical Sciences* **2008,** *11* (1), 167–177.
2. Son, G.-H.; Lee, B.-J.; Cho, C.-W., Mechanisms of drug release from advanced drug formulations such as polymeric-based drug-delivery systems and lipid nanoparticles. *Journal of Pharmaceutical Investigation* **2017,** *47* (4), 287–296.
